# Common Transcriptional Program of Liver Fibrosis in Mouse Genetic Models and Humans

**DOI:** 10.3390/ijms22020832

**Published:** 2021-01-15

**Authors:** Kaja Blagotinšek Cokan, Žiga Urlep, Miha Moškon, Miha Mraz, Xiang Yi Kong, Winnie Eskild, Damjana Rozman, Peter Juvan, Tadeja Režen

**Affiliations:** 1 Centre for Functional Genomics and Bio-Chips, Institute of Biochemistry and Molecular Genetics, Faculty of Medicine, University of Ljubljana, 1000 Ljubljana, Slovenia; kaja.blagotinsek@mf.uni-lj.si (K.B.C.); zigaurlep@gmail.com (Ž.U.); damjana.rozman@mf.uni-lj.si (D.R.); peter.juvan@mf.uni-lj.si (P.J.); 2 Computational Biology Group, Faculty of Computer and Information Science, University of Ljubljana, 1000 Ljubljana, Slovenia; miha.moskon@fri.uni-lj.si (M.M.); miha.mraz@fri.uni-lj.si (M.M.); 3 Research Institute of Internal Medicine, Oslo University Hospital Rikshospitalet, 0372 Oslo, Norway; x.y.kong@medisin.uio.no; 4 Section for Biochemistry and Molecular Biology, Department of Biosciences, Faculty of Mathematics and Natural Science, University of Oslo, 0315 Oslo, Norway; winnie.eskild@ibv.uio.no

**Keywords:** NAFLD, NASH, bile acid, GEM, fibrosis, fatty acid

## Abstract

Multifactorial metabolic diseases, such as non-alcoholic fatty liver disease, are a major burden to modern societies, and frequently present with no clearly defined molecular biomarkers. Herein we used system medicine approaches to decipher signatures of liver fibrosis in mouse models with malfunction in genes from unrelated biological pathways: cholesterol synthesis—*Cyp51*, notch signaling—*Rbpj*, nuclear factor kappa-light-chain-enhancer of activated B cells (NF-κB) signaling—*Ikbkg*, and unknown lysosomal pathway—*Glmp*. Enrichment analyses of Kyoto Encyclopedia of Genes and Genomes (KEGG), Reactome and TRANScription FACtor (TRANSFAC) databases complemented with genome-scale metabolic modeling revealed fibrotic signatures highly similar to liver pathologies in humans. The diverse genetic models of liver fibrosis exposed a common transcriptional program with activated estrogen receptor alpha (ERα) signaling, and a network of interactions between regulators of lipid metabolism and transcription factors from cancer pathways and the immune system. The novel hallmarks of fibrosis are downregulated lipid pathways, including fatty acid, bile acid, and steroid hormone metabolism. Moreover, distinct metabolic subtypes of liver fibrosis were proposed, supported by unique enrichment of transcription factors based on the type of insult, disease stage, or potentially, also sex. The discovered novel features of multifactorial liver fibrotic pathologies could aid also in improved stratification of other fibrosis related pathologies.

## 1. Introduction

Fibrosis is a common feature of skin, lung, kidney, and liver diseases; however, it can affect virtually every organ. It is characterized by excessive deposition of connective tissue components, which leads to tissue remodeling and organ malfunction. High mortality is associated with fibrotic diseases. The progress in development of anti-fibrotic drugs is slow, especially for individual fibrotic diseases where mechanisms are not clear. There is a need to unravel the core fibrotic pathways across different fibrotic diseases, as well as across the same type of fibrotic disease that can arise from a multitude of causes. It is believed that, in addition to common fibrotic programs, other factors influencing fibrotic disease susceptibility may be distinct, with disease-specific and organ-specific risk factors [1].

Liver fibrosis is a characteristic of the progressive liver pathologies defined by accumulation of collagen, smooth-muscle actin, hydroxyproline, etc., and is one of the hallmarks of the advanced stages of non-alcoholic fatty liver disease (NAFLD), currently called metabolism-associated fatty liver disease (MAFLD). This is a multifactorial disease with variable etiology and no clearly defined molecular biomarkers for diagnosis, prognosis, or progression [2]. The advanced disease stages include non-alcoholic steatohepatitis (NASH) and cirrhosis, both potentially leading to liver cancer. The prevalence and hence the burden of the disease is increasing because of the lack of approved pharmacotherapies and low impact of prevention strategies [3]. In animal models, liver fibrosis is a result of a chronic liver injury induced by different factors, which range from alcohol, diets, toxins, drugs, bile duct ligation, genetic modifications, and others. Each type of liver injury activates a specific program at cellular and molecular levels [4], and if sustained, disease progresses to further stages [5].

In humans, NAFLD and NASH have many faces, as clinical manifestations are highly heterogeneous [6]. Cirrhosis may or may not be present; not all patients show abnormal blood parameters; comorbidities, such as diabetes and obesity, vary, and the presence of fibrosis and steatosis is not uniform. Clinical drug trials consistently show that targeting NAFLD histological features does not always result in disease resolution. For example, reduction of steatosis did not improve other histological outcomes of NAFLD [7] and elevated steatosis did not always associate with worsening of fibrosis [8]. All of this indicates that there are potentially different subtypes of NAFLD patients. In concordance, a recent study identified three NAFLD subtypes in relation to methionine/folate cycle, according to serum metabolite signature, also predicting the progression to NASH [9]. The latest recommendation was a subcategorization of NASH patients to identify those who will be best suited for specific treatments in clinical trials [8].

As resolution of fibrosis is one of the endpoints of clinical trials, we can benefit from a variety of mouse models that develop progressive fibrosis similarly to humans. In our previous work, we discovered that hepatocyte-specific *Cyp51* (cytochrome P450 or lanosterol 14α-demethylase) knockout (LKO) males and females develop liver fibrosis (without steatosis or cholestasis) due to blockage of cholesterol synthesis [10]. Among metabolic alterations were: deregulated sterol intermediates, decrease in hepatic cholesterol and its esters, modified bile acid composition, and elevated plasma total cholesterol and high-density lipoprotein (HDL) in a sex-specific manner. Similarly, the whole-body knockout (KO) of *Glmp* (glycosylated lysosomal membrane protein) presented with liver fibrosis although the function of this lysosomal protein, remains to be clarified [11]. Among metabolic alterations in *Glmp* KO mice are increased liver bile acids and infiltration of inflammatory cells [12]. Decreased blood glucose, triglycerides (TAG) and non-esterified fatty acids were also observed, together with increased liver TAGs, although liver steatosis was not confirmed histologically [13].

As two such different KO models both result in a similar liver phenotype, we hypothesized that it might be possible to determine a common fibrotic signature from multiple mouse models. We focused on single gene knockouts that develop histologically confirmed liver fibrosis (with or without steatosis or cholestasis) without additional dietary or chemical insults, preferably in both sexes, and with well annotated transcriptome data. In addition to *Cyp51,* LKO, and *Glmp* KO, the fibrotic phenotype also develops in the liver knockout of a notch signaling pathway repressor *Rbpj* (recombination signal binding protein for immunoglobulin kappa J region) due to impaired bile duct maturation causing obstructive bile acid flow, accumulation of bile acids, necrosis, and severe cholestasis with progression to hepatocellular carcinoma (HCC) [14]. Fibrosis is also a feature of the hepatocyte-specific *Ikbkg* (*Nemo*, Inhibitor of kappa B kinase gamma) knockout males with steatohepatitis and HCC through changing the response to inflammation [15]. Increased oxidative stress was present, mitochondria appeared to be affected, irregular glycogen deposits were observed, and serum glucose was decreased while serum TAG and cholesterol levels were unchanged [16].

We aimed to study the fibrotic signatures of both sexes; however, only the *Cyp51* LKO transcriptome data also include females. Using functional comparative analysis of gene expression based on Kyoto Encyclopedia of Genes and Genomes (KEGG), Reactome and TRANScription FACtor (TRANSFAC) databases, as well as genome-scale metabolic models (GEMs), we could identify multiple common fibrotic transcriptome signatures, with high similarity to human NAFLD and NASH. The hallmark is downregulation of metabolic pathways and upregulation of immune system-related pathways and pathways in cancer. We provide also new insights into the function of GLMP in the liver and propose “universal” fibrosis-related biomarkers with some sex dependencies.

## 2. Results

### 2.1. Similar Transcriptome Alterations Caused by Different Genetic Defects

**Figure 1:**
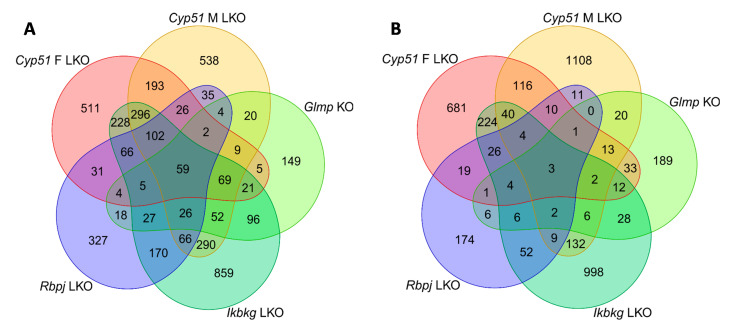
Venn diagrams of differentially expressed genes (DEGs) in mouse genetic models of liver fibrosis. (**A**) Upregulated DEGs. (**B**) Downregulated DEGs.

**Figure 2:**
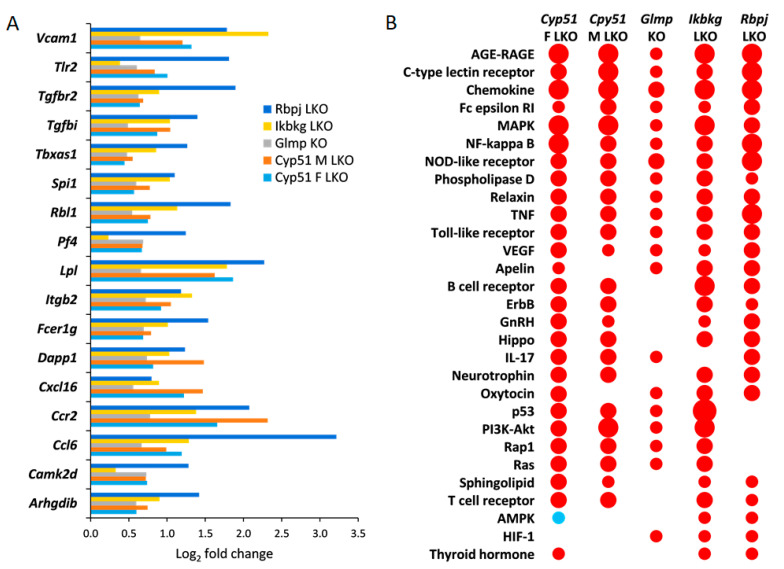
Common significantly enriched Kyoto Encyclopedia of Genes and Genomes (KEGG) signaling pathways and DEGs. (**A**). Log_2_ fold change in expression of selected common DEGs in all fibrotic models. Only DEGs, which are a part of common enriched signaling or metabolic KEGG pathways, are presented. Log_2_ fold change represents log_2_ ratio between average knockout (KO) vs. wild type (WT). (**B**). KEGG signaling pathways common to at least three mouse genetic models of liver fibrosis as calculated using pGSEA (Parametric gene set enrichment analysis)are presented. Red indicates positive enrichment and blue negative. The size of the bubble reflects the fold change of each pathway.

The characteristics of genetic mouse models are summarized in Table S1. They were all adult males with C57BL/6J genetic background, in addition to a group of *Cyp51* LKO females, with histologically confirmed fibrosis, increased inflammation (except *Rbpj* LKO where it was not measured), and final progression to liver tumors or dysplastic nodules. The *Cyp51* LKO and *Glmp* KO did not present with histological steatosis and cholestasis. We analyzed the liver transcriptome data and compared the differentially expressed genes (DEGs) between these genetic models of fibrosis (Table S2). At the intersection, we observed a higher number of upregulated (59) than downregulated genes (3) (Figure 1). A higher percentage of upregulated genes was common also when we compared the overlap of DEGs between each pair of fibrotic models (Table S3). Clustering analyses of 62 common DEGs showed that the most similar models are *Cyp51* F and M LKO and *Ikbkg* LKO (Figure S1). From the common DEGs, the majority have a function at the plasma membrane or extracellular space or are involved in immune response pathways, among them *Tgfbr2, Tgfbi* from TGF-β (Transforming growth factor beta) signaling, and the lipoprotein lipase (Figure 2A). TGFBI (Transforming growth factor beta induced) was recently confirmed to be strongly associated with NAFLD and cirrhosis in humans [17]. These results lead us to a conclusion that the common transcriptional program of liver fibrosis is largely represented in the set of upregulated genes, while downregulation of genes is more associated with the unique program probably related to the type of the insult, disease stage or sex. Additionally, the overlap in DEGs is affected by different biological variables of the models (age and stage of the disease). We could not exclude effects of technical factors, such as RNA isolation, type of microarrays, etc. Therefore, we focused on pathway analyses, which are less sensitive to such effects compared to expression of individual genes.

### 2.2. Common KEGG and Reactome Pathways in Different Fibrotic Models

Positively enriched KEGG (65) and Reactome (52) pathways common to all liver fibrotic models have roles in response to liver injury, repair, hemostasis, cancer, regulation of metabolism, development of fibrosis, and activation of the immune system (Tables S4 and S5). Both pathway analyses confirmed that models are most similar in positively enriched pathways (Table S3). At the intersection of different genetic models, there were many positively enriched KEGG pathways indicating an activation of a common transcriptional program in fibrosis regardless of the type of injury, age, or sex (Figure 2B). Common enriched Reactome and KEGG pathways were, with few exceptions, always enriched in the same direction in the models. One exception was AMPK (AMP-activated protein kinase) signaling, which was positively enriched in *Ikbkg* LKO and *Rbpj* LKO, but negatively in female *Cyp51* LKO (Figure 2B). Pathway analysis revealed that cellular organelles are affected during progression of fibrosis. For example, peroxisome pathways (Peroxisomal protein import, Peroxisome pathway) and Autophagy were negatively enriched while Lysosome was enriched positively. Interestingly, with exception of *Glmp* KO and the male *Cyp51* LKO, HCC pathway was enriched positively, indicating an early commitment of liver cells towards cancer in these models.

### 2.3. Mouse Genetic Models Show Overlapping Transcriptome Signatures with Human NAFLD and NASH

It is important to compare the mouse genetic models of hepatic fibrosis to human NAFLD and NASH. We selected a list of enriched KEGG pathways calculated by GSEA (Gene set enrichment analysis) in patients with NAFLD (N = 27) and NASH (N = 25) as presented in Teufel et al. [18], where liver transcriptome data from NAFLD and NASH patients of both sexes were compared to control (N = 39) and healthy obese (N = 25) patients and also to mouse female diet models. In our analyses, we used the Teufel data for direct comparison of our pGSEA (Parametric gene set enrichment analysis) analyses from genetic models with data from patients with NAFLD and NASH (Table S6). There were many common, positively enriched KEGG signaling pathways between mouse genetic models and human NAFLD and NASH, which is in line with a common fibrotic program (Table 1). Interestingly, the genetic fibrotic models had a much higher overlap of enriched KEGG pathways with human NAFLD and NASH compared to dietary mouse models reported in Teufel et al. [18]. This was surprising since in humans, diet is supposed to be a crucial factor in the majority of NAFLD cases. However, we cannot exclude that some of the observed distinctions were not due to differences between humans and mice or due to diet, but might be linked to sex. Our mouse models were of both sexes while Teufel data included NAFLD and NASH patients of both sexes and only female dietary mouse models.

### 2.4. Negative Enrichment of Metabolic Pathways in Fibrosis

We discovered that negative enrichment of metabolic pathways is a hallmark of fibrosis. From basic metabolism, bile and fatty acids (linoleic), steroid hormone, ketone, butanoate, nitrogen, heme, and branched-chain amino acid-related KEGG and Reactome pathways were enriched negatively in the four genetic fibrotic models (Table 2, Tables S4 and S5). Most importantly, the upstream pathways regulating metabolism were also negatively enriched, such as several nuclear receptors, IGF1R (Insulin like growth factor 1 receptor) and insulin signaling (Table S5). In contrast, Glycosphingolipid and Sphingolipid metabolism and its signaling were positively enriched in all models except *Glmp* KO. These results indicate that the modulation of metabolic pathways happens regardless of the type of injury, metabolic state, or sex, and also in absence of dietary manipulation.

Focusing on more unique pathways and DEGs that are not common among models, we observed distinct changes in metabolism of almost all amino acids, carbohydrates, vitamins, cofactors, and energy metabolism, exposing female *Cyp51* LKO and male *Ikbkg* LKO models as the most affected (Table 2). These results propose specific metabolic programs and the existence of different metabolic subtypes depending on the type of injury, stage of fibrosis or sex. In conclusion, data from genetic fibrotic models exposed a wide array of metabolic rearrangements as the hallmark of the fibrosis program.

### 2.5. Genome-Scale Metabolic Models Confirmed Rearrangements in Lipid Metabolism Pathways

**Figure 3:**
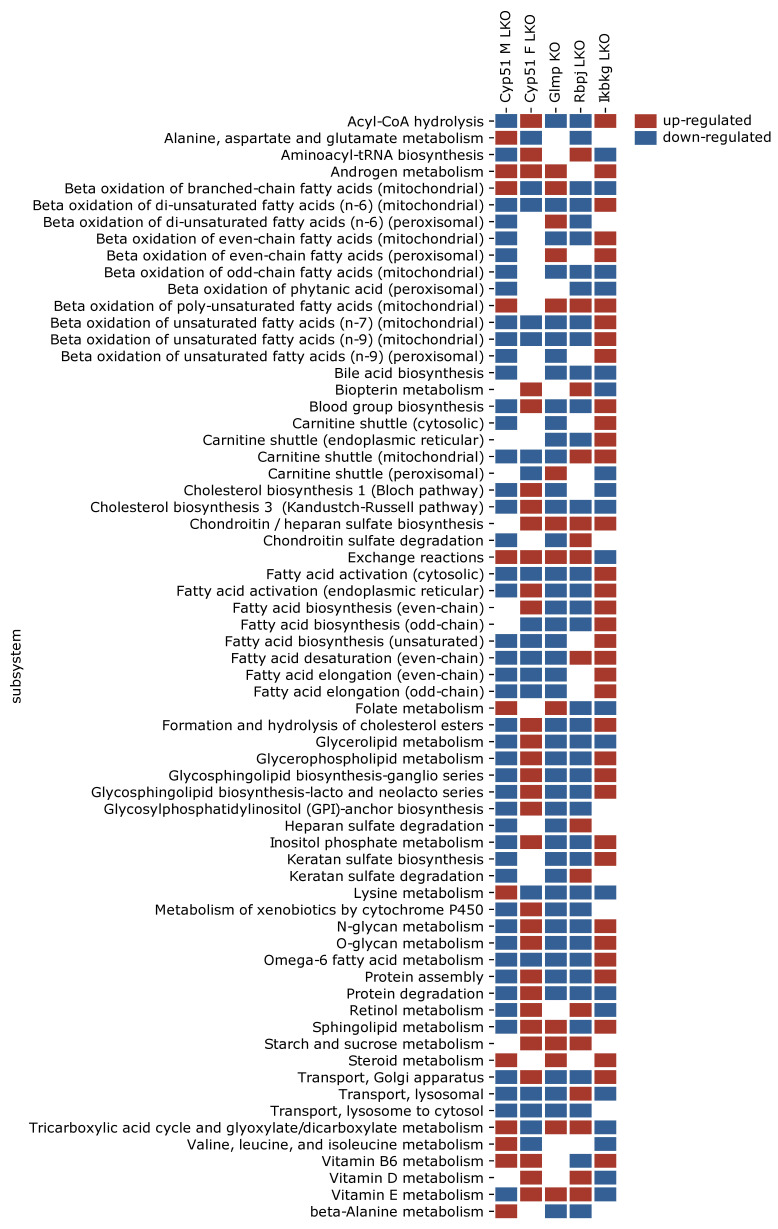
Statistically significantly perturbed genome-scale metabolic model (GEM) subsystems. Presented are subsystems common to at least three models of liver fibrosis. Red is upregulated, blue is downregulated.

Since metabolic rearrangements were enriched in pathway analyses, we used GEMs to simulate and predict metabolic fluxes at systems-level using transcriptome data. Figure 3 represents statistically significant changes in GEMs common to at least three models, with the majority involved in fatty acids metabolism (synthesis, oxidation, transport) in different cellular compartments (cytosol, mitochondria, peroxisome). Importantly, several sterol-related GEMs were affected by fibrosis, such as cholesterol and bile acid synthesis, cholesterol esters and steroid metabolism (Figure 3). The levels of liver cholesterol, cholesterol esters and bile acids were decreased in the *Cyp51* M LKO model, indicating that similar conditions might be present in other genetic models [10]. Several other lipid pathways were rearranged during fibrosis, such as glycerolipid, glycerophospholipid and sphingolipid pathways. GEM analyses also detected changes in carnitine shuttle, transport reactions and retinol metabolism. Again, each model exhibited a unique combination of metabolic rearrangement. Importantly, the GEM analyses confirmed the global rearrangement of lipid homeostasis as a part of the common program in liver fibrosis. Moreover, GEM analyses indicated that the type of injury defined which cellular compartment was affected and in what way.

### 2.6. Enrichment of Transcription Factors Exposed Variability in Metabolic Regulators

**Figure 4:**
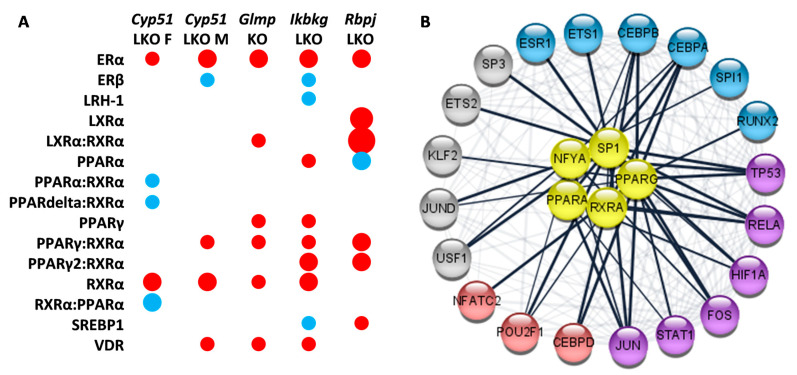
Selected enriched transcription factors (TFs) in mouse genetic models of liver fibrosis. (**A**). Model-specific enrichment of nuclear receptors in fibrotic models. Red indicates positive enrichment and blue negative. The size of the bubble reflects the fold change of each pathway. (**B**). Common enriched transcription factors reveal network-like interactions between regulators of lipid metabolism (yellow) and TFs involved in regulation of cancer pathways (blue and violet) and the immune system (red and violet).

Transcription factors (TFs) are upstream regulators of metabolism and are thus upstream of metabolic pathways in KEGG and Reactome, adding another level of understanding. The majority of TFs were positively enriched (Tables S3 and S7). Twenty TFs were enriched in all genetic fibrotic models, among them estrogen receptor alpha (ERα), NF-Y, c-ETS-1, and an additional 16 were common to four S7). All were enriched positively and involved in the regulation of the immune system, cancer, or metabolic or hormone pathways. Several nuclear receptors regulating lipid metabolism and estrogen receptor α were enriched, coinciding with enrichment of Reactome pathways related to estrogen receptor and nuclear receptors (Figure 4A). Cytoscape analysis using the STRING database revealed a network of interactions between the common TFs regulating lipid metabolism and common TFs regulating cancer pathways and the immune system (Figure 4B). It is important to note that the enrichment of TFs regulating lipid metabolism varied among the studied genetic models (Figure 4A). For example, E2F-1 (E2F transcription factor 1), a mediator of sustained lipogenesis and contributor to hepatic steatosis, was enriched positively in *Ikbkg* LKO and *Rbpj* LKO, and negatively in *Glmp* KO, coinciding with histological findings. PPARγ:RXRα (Peroxisome proliferator activated receptor gamma: Retinoid X receptor alpha), RXRα, and VDR (Vitamin D receptor) were positively enriched in at least three models, LXRα (Liver X receptor alpha) and PPARα (Peroxisome proliferator activated receptor alpha) in two to three, while FXR (Farnesoid X receptor) was not enriched at all. Another known lipid regulator is SREBP1 (Sterol regulatory element-binding protein 1), which was positively enriched in *Rbpj* LKO, and negatively in *Ikbkg* LKO. These data indicate that the combination of enriched TFs, the regulators of metabolism, could depend on genetic background and could be used to predict the metabolic subtypes of fibrosis.

## 3. Discussion

While it is believed that fibrosis can arise through a multitude of causes, it is nevertheless reasonable to believe that a common “fibrotic program” is hidden beneath that. To address this hypothesis, we used systems medicine approaches and pathway analyses to decipher transcriptome signatures of four genetic mouse models of liver fibrosis, one available in both sexes. In each of these models, a single gene has been knocked out and no dietary or chemical manipulation was used. Malfunction of genes with very different biological roles (cholesterol biosynthesis, notch and nuclear factor kappa-light-chain-enhancer of activated B cells (NF-κB) signaling, and unknown lysosomal membrane protein) resulted in liver fibrosis, which progressed to liver cancer.

**Figure 5:**
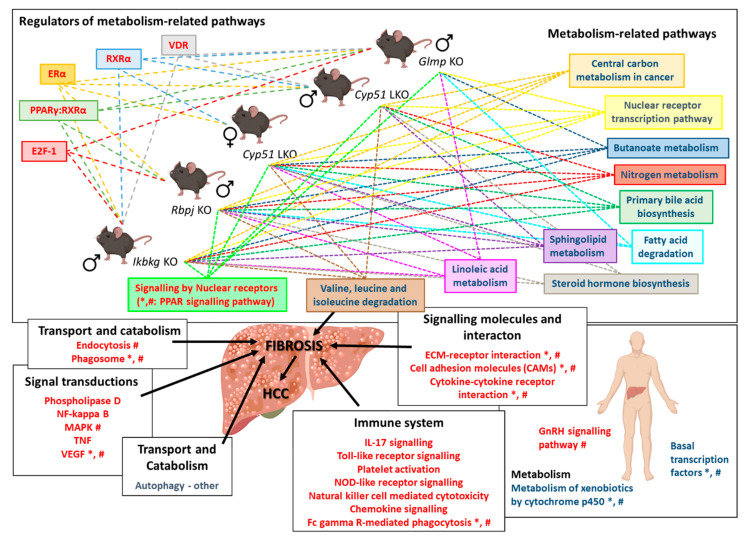
Scheme of the common transcriptional program in mouse liver fibrotic models. Knocking out a single gene from different unrelated pathways (*Cyp51*, *Rbpj*, *Ikbkg,* and *Glmp*) leads to downregulation of metabolism-related pathways, regulated by different TFs, and upregulation of signaling pathways, resulting in fibrosis. Mouse transcriptome data from mouse models was compared to human NAFLD and NASH transcriptome data [18]. *—pathways/TFs enriched also in human NAFLD; ^#^—pathways/TFs enriched in human NASH. Blue text color—negatively enriched KEGG/Reactome/TFs; Red text color—positively enriched KEGG/Reactome/TFs. Dashed arrows indicate enriched pathways/TFs in individual liver fibrotic mouse model. Icons are used from the BioRender library.

Herein we show that despite the different genetic insults, different sex, age, and the disease stage, a common fibrotic transcriptional program was identified (Figure 5). Positively enriched KEGG and Reactome pathways were predominantly involved in the immune system, extracellular matrix, cell-cell communication, hemostasis, and cancer. This common program is very similar to human NAFLD and NASH [18]. Downregulation of fatty acid metabolism and positive enrichment of platelets and hemostasis-related pathways is a hallmark of our data as well as transcriptomes of human NASH [19,20,21,22,23].

**Figure 6:**
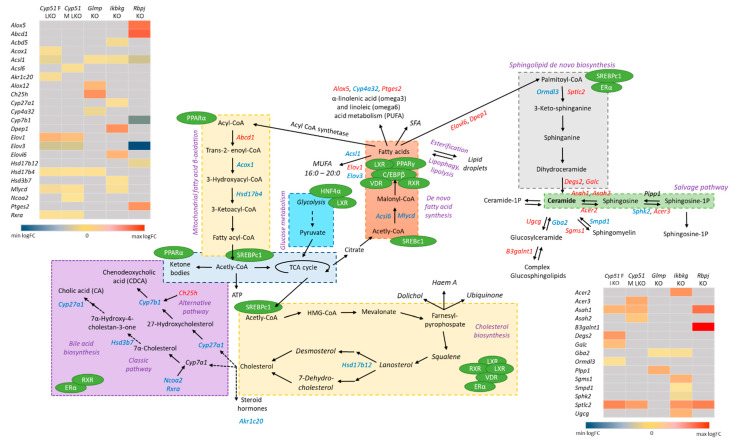
Unique lipid metabolite’s rearrangements in mouse liver fibrotic models. Pathways analyses and GEM subsystems detected model-specific deregulation of lipid metabolism in genetic models. Blue color indicates downregulated DEGs, while red are upregulated DEGs. Grey in heat maps represents insignificant expression. Green circles are detected TFs in liver mouse models, which regulate different metabolic pathways (violet). Legend of heat maps are shown at the bottom.

The negative enrichment of liver metabolic pathways indicates a molecular link between disrupted energy homeostasis and cell cycle control, which could be crucial for the development of NASH-related HCC [24]. Pathway analyses and GEMs detected wide rearrangements in the metabolism of sphingolipids, ketones, bile, linoleic, and fatty acids, as well as branched amino acids in all genetic models of fibrosis. These groups of metabolites present potential serum biomarkers of NAFLD/NASH progression since the changes seem to be independent of the etiology of the disease. Furthermore, they represent potential new diagnostic and prognostic biomarkers of liver diseases in humans (Table 3). Bile acids are an example of a potential common serum biomarker of liver diseases. A potential prognostic biomarker was identified by a metabolomics prospective study where serum fatty acids, including linoleic and α-linoleic acids, were lower before the occurrence of cirrhosis in patients in comparison to healthy controls [25]. The observed model-specific changes in transcriptome signatures could reflect the unique metabolic rearrangements among fibrotic models (Figure 6). For example, the female *Cyp51* LKO model has upregulated genes, indicating increase in GM2 ganglioside and decrease in GM3 ganglioside, while the male *Cyp51* LKO, *Ikbkg* LKO and *Rbpj* LKOs have potentially increased GM3 ganglioside. Overall, it seems very plausible that serum metabolites reflect the stage of liver disease and the patient’s metabolic state, and could enable differentiation of metabolic subtypes of NAFLD, NASH, and beyond. For example, C4 (7-alpha-hydroxy-4-cholesten-3-one), a bile acid intermediate used to assess liver bile acid biosynthesis, was increased in obese NAFLD patients [26], but decreased in lean patients [27]. A combination of serum metabolites could be used for patient stratification in personalized medicine. This is further supported by the fact that also in genetic models of liver fibrosis significant changes in blood and liver metabolites were observed [10,12,13,16,28]. For example, bile acids were increased in serum of *Glmp* KO, while they were decreased in bile in *Cyp51* F and M LKO. A decrease in blood TAG and non-esterified fatty acids was observed in *Glmp* KO, no change was observed in blood TAG in *Ikbkg* LKO model, while liver TAG were increased in both models. A decrease in liver total cholesterol was observed only in *Cyp51* M LKO, while esterified liver cholesterol was decreased in *Cyp51* F and M LKO. Plasma total and LDL cholesterol were increased in *Cyp51* M LKO, while no changes were observed in *Cyp51* F LKO and *Ikbkg* LKO model. A decrease in blood glucose was observed in *Glmp* KO and *Ikbkg* LKO, while in liver, glycogen deposits accumulated in the Ikbkg LKO model. More importantly, we emphasize that these genetic models develop metabolic rearrangements similar to NAFLD and NASH without obesity, dietary or chemical manipulation. We propose that overall metabolic rearrangements are crucial for the “fibrotic transcriptional program”. However, type of the injury, stage of fibrosis and sex, define the direction, degree and type of metabolic pathway affected.

Applying different approaches and databases enabled a fresh perspective on the fibrotic transcriptome data, also allowing us to consider transcription factors as major regulators of cellular metabolism. The Reactome pathways nuclear receptor transcription pathway and regulation of lipid metabolism by peroxisome proliferate-activated receptor-α were negatively enriched, while signaling by nuclear receptors and extra-nuclear estrogen signaling were positively enriched. This underlines the importance of transcriptional reprogramming of metabolism in fibrosis. Recent studies exposed a suppression of liver-identity transcription factors induced by liver injury [51]. Two models, female *CYP51* LKO and *Ikbkg* LKO, exhibit a suppression of these TFs. Furthermore, several TFs regulating the lipid metabolism were enriched, such as PPARγ, RXRα, VDR, PPARα, SREBF1C, and LXR. Their expression is affected by NAFLD or NASH progression in human livers (Table 3). However, enrichment of these TF was not overlapping among genetic models, indicating that regulation of metabolism is specific to each genetic model of fibrosis and also defines the manner of metabolic rearrangements. For example, female *CYP51* LKO has the strongest inhibition of overall metabolism, including the TCA cycle, and fatty acid and amino acid metabolism, while it is also the only model with a negative enrichment of metabolic regulators AMPK, PPARδ and PPARα. The different transcriptome landscapes resulting from different genetic backgrounds could be considered as stratified metabolic subtypes of NAFLD or NASH. This view may help to explain the variable success of treatments targeting these metabolic regulators [6]. Based on this, we propose that regulators and their downstream metabolites that differ between the genetic models warrant further testing as potential biomarkers in a human setting, to enable stratification of patients for metabolic subtypes of fibrosis. This could substantially increase the impact of existing and novel therapeutic strategies.

Epidemiological data in humans clearly show that estrogen has a protective role against NAFLD and NASH in premenopausal females [52]. Thus, sex hormones affect the development of liver diseases [53]. Our pathway and TF enrichment analyses and GEMs exposed changes in overall steroid hormone metabolism as one of the hallmarks of the fibrotic program. Extra-nuclear estrogen signaling and ERα (Estrogen receptor alpha) were positively enriched in all mouse models, regardless of sex, while Steroid hormone biosynthesis was negatively enriched in four models. Data in humans confirm increased expression of ERα in NAFLD livers and its correlation with the severity of steatosis [54]. ERα knockout in mice present with induced steatosis in both sexes, indicating that ERα activation in fibrosis could be a sex-independent protective adaptation against liver insults, exposing estrogen receptor as a potential drug target for NAFLD management [55,56,57]. Interestingly, even though we have previously shown a sex-dependent difference in the progression of liver fibrosis [58], we observe similar changes in steroid-related pathways in both sexes [10]. Based on our analysis we cannot draw conclusions regarding fibrotic programs in each sex since too little data is available for the females. Signaling through the estrogen receptor seems to be a part of the common fibrotic transcriptional program, regardless of the insult or sex. An important aspect of stratification could be potential differences in the liver fibrotic transcriptome signatures between females and males. It will therefore be important to have more comparable data available for both sexes.

Since one of the fibrotic models has a knockout in the gene *Glmp*, a gene whose function is not fully understood, the comparative transcriptome analysis helped in shedding light on its role at the molecular level. The full knockout of GLMP leads to liver fibrosis with inflammation, oval cell activation, and proliferation, hepatocyte apoptosis, oxidative stress, and development of HCC and hemangioma-like tumors from the age of 12 months [11,12]. Since evidence for HCC was not substantiated in KEGG analyses, in contrast to other mouse models, we anticipate that alternative pathways are responsible. A likely explanation for liver cancer in *Glmp* KO mice is impaired autophagy due to deficiency of lysosomes, which may on one hand contribute to the pathogenesis of NAFLD [59], and can also lead to lysosomal storage disorders [60]. A recent report demonstrated that Major Facilitator Superfamily Domain Containing 1 (MFSD1) and GLMP, both lysosomal membrane proteins, interact and affect each other’s expression [61]. MFSD1 belongs to a group of proteins transporting nutrients, waste, and ions across membranes [62]. A dysfunctional GLMP/MFSD1 complex could induce abnormal functions in lysosomes, severely affecting autophagy [63]. We propose that disturbances in these two pathways work cooperatively in increasing ER stress, the inflammatory-related KEGG pathway and development of fibrosis [64]. This is corroborated by the observed negative enrichment of mTOR (The mammalian target of rapamycin) signaling and KEGG pathways associated with detoxification, such as cytochromes P450.

## 4. Materials and Methods

### 4.1. Microarray-Based Gene Expression Analysis

*Glmp* KO and *Cyp51* LKO transcriptomes were determined in-house and deposited in GEO, while raw transcriptome data for *Ikbkg* LKO and, *Rbpj* LKO models were obtained from GEO. The animal experiments, ethical statements and details about RNA isolation from the liver of Glmp KO and Cyp51 LKO genetic models are described in the original papers [10,13]. To assess the *Glmp* transcriptome, we used total RNA isolated as described in Kong X.Y. et al. 2015 [13]. We hybridized Affymetrix GeneChip Mouse Gene 2.0 ST Arrays (Affymetrix, Santa Clara, CA, USA) with samples from livers of 16 *Glmp KO* and *Glmp* wild type (WT) mice at age 8 and 18 weeks. Each group consisted of 4 samples (Table S8). Data analyses were performed using R and Bioconductor software packages (https://www.bioconductor.org/). We normalized raw (CEL) expression data using the RMA algorithm from the *oligo* package [65]. Quality control and outlier detection were performed using the arrayQualityMetrics package before and after normalization [66]. Raw as well as normalized data were deposited in GEO under the accession GSE154021.

The generation of transcriptome data from 19-week *Cyp51* LKO (GSE58271), 4-week *Rbpj* LKO (GSE121302), 8- to 9-week *Ikbkg* LKO (GSE33161) and resulting mice were described previously [10,14,28]. The RMA algorithm from the *oligo* package and quantile normalization from the *limma* package [67] were used for re-normalization of raw expression data from Affymetrix and Agilent arrays, respectively. *limma* was used to fit individual normalized gene expression data using linear regression models as shown in Table S8. Empirical Bayes statistics were used to estimate the statistical significance of expression differences of genes and the Benjamini–Hochberg procedure was used to calculate false discovery rate (FDR) of differential expression. For selecting DEGs, a FDR cut-off at α < 0.05 was used, no log fold change cut-off was applied.

KEGG pathways [68], Reactome pathways [69] and TRANSFAC database version 2020.1 [70] were used for functional enrichment studies. Gene sets containing 5 or more elements were constructed and tested for enrichment using the PGSEA package [71]. In the case of TF enrichment, factors were merged based on their ID irrespective of their binding sites. Statistical significance of gene set enrichment was estimated using the same approach as for individual genes.

To facilitate comparative functional genomics analysis, DEGs and enriched gene sets were partitioned according to their overlaps between the studies. Genes and gene sets were split into up/down regulated and positively/negatively enriched groups and their numbers are reported. Overlaps between the models are visualized by Venn using the VennDiagram package [72]. For hierarchical clustering of samples/genes and heat map visualization, gene expression was scaled per sample to have mean zero and standard deviation one.

Functional similarity between the mouse models was quantified as a ratio of significant expression/enrichment changes that are in common to the models vs. the significant expression/enrichment changes of each model individually, e.g., the number of DEGs in the intersection of two models was divided by the number of DEGs for each model. Thus, a non-symmetric similarity matrix was calculated summarizing similarities between all pairs of models from a perspective of each model. Ratios of significant DEGs are shown in Table S3, together with the number of DEGs. Furthermore, the similarity between models is expressed separately for positive and negative expression/enrichment changes; thus each pair of the model is characterized by two ratios, left for positive and right for negative expression changes. Ratios are represented row-wise: the number of DEGs in common was divided by the number of DEGs of the model within the corresponding row. Table S3 show similarities between the models for KEGG and Reactome pathways and TFs, respectively.

### 4.2. Genome–Scale Metabolic Modeling

We performed the integration of DEGs into the GEM of C57BL6/J mice liver tissue, which was previously described [20] and is available in the Metabolic Atlas Database (www.metabolicatlas.org) [73]. DEGs were integrated into the model using the Metabolic Adjustment by Differential Expression (MADE) method [74,75]. MADE integrates differential expression data into a reference model using flux balance analysis (FBA) to obtain a functional metabolic model describing a perturbed state of a system (e.g., after gene silencing). When reference and perturbed models are available, up-/down-regulated reactions can be identified using the flux variability analysis (FVA) [76]. The list of up-/down-regulated reactions obtained with the FVA was used to perform metabolic subsystem enrichment analysis based on the hypergeometric test. The Benjamini and Hochberg procedure was used for *p* value adjustment. The cut-off value for significantly up-/down-regulated subsystems was set to 0.05.

## 5. Conclusions

Based on comparing different genetic models of liver fibrosis without dietary manipulation, we revealed common liver fibrotic transcriptome signatures with high similarity to signatures of human NAFLD and NASH. A hallmark of the fibrotic program are changes in metabolic pathways related to lipids, such as bile acids, steroids, sphingolipids, and fatty acids. These metabolites and their regulators (AMPK, FOXOA1, SREBP1, LXRα, PPARδ, and PPARα) exhibit enrichment that depends on the genetic background, exposing their potential to serve as diagnostic and prognostic biomarkers of fibrotic subtypes also in humans. They could also enable a more precise metabolism-related stratification of NAFLD/NASH patients before entering clinical drug trials and facilitate the implementation of personalized liver disease management.

## Figures and Tables

**Table 1:** Top enriched KEGG pathways common between genetic mouse models of liver fibrosis and human non-alcoholic fatty liver disease (NAFLD) and non-alcoholic steatohepatitis (NASH) from Teufel et al. [18].

KEGG Pathway	NAFLD	NASH	*Cyp51* F LKO	*Cyp51* M LKO	*Glmp* KO	*Ikbkg* LKO	*Rbpj* LKO
Antigen processing and presentation	+	+	1.00	ns	0.84	1.05	ns
B cell receptor signaling pathway	+	+	0.89	0.77	ns	1.06	0.91
Cell adhesion molecules (CAMs)	+	+	1.43	1.12	1.02	1.34	0.60
Cell cycle	ns	+	1.25	ns	0.50	2.40	1.40
Chemokine signaling pathway	ns	+	1.06	0.78	0.89	1.17	1.39
Colorectal cancer	ns	+	0.87	0.73	ns	0.84	0.47
Cytokine–cytokine receptor interaction	ns	+	0.76	0.77	0.71	0.93	1.21
DNA replication	+	+	0.99	ns	ns	1.01	1.23
ECM-receptor interaction	+	+	1.67	1.87	0.54	1.50	0.50
Endocytosis	ns	+	1.20	0.72	ns	0.93	0.65
ErbB signaling pathway	ns	+	0.80	0.56	ns	0.78	0.49
Fc epsilon RI signaling pathway	+	+	0.67	0.40	ns	0.50	0.75
Fc gamma R-mediated phagocytosis	+	+	1.08	0.93	ns	1.06	0.97
Focal adhesion	+	+	1.96	1.85	ns	1.65	0.67
Hematopoietic cell lineage	+	+	1.20	1.59	1.23	1.05	0.95
Leukocyte transendothelial migration	+	+	1.52	1.11	0.70	1.08	0.90
MAPK signaling pathway	ns	+	1.09	1.08	ns	1.26	0.70
Natural killer cell mediated cytotoxicity	ns	+	0.95	ns	0.54	0.87	0.80
Neurotrophin signaling pathway	ns	+	0.98	0.53	ns	0.63	0.60
Pancreatic cancer	+	+	1.01	0.76	ns	0.95	0.42
Pathways in cancer	ns	+	1.42	1.18	ns	1.31	1.21
Phagosome	+	+	1.71	1.48	1.10	1.70	1.53
Regulation of actin cytoskeleton	ns	+	1.77	1.39	ns	1.27	0.63
Small cell lung cancer	+	+	1.21	1.18	0.44	1.02	0.64
T cell receptor signaling pathway	ns	+	0.73	0.52	ns	0.63	0.40
Toll-like receptor signaling pathway	ns	+	0.85	0.70	ns	0.69	0.65
VEGF signaling pathway	+	+	0.48	0.28	ns	0.49	0.54

Presented are log_2_ fold changes comparing KO vs. WT for mouse models and direction of enrichment for human NAFLD and NASH data, where + means enrichment and ns—not significant.

**Table 2:** Selected statistically significantly enriched Reactome pathways from metabolism.

Reactome Pathway	*Cyp51* F LKO	*Cyp51* M LKO	*Glmp* KO	*Ikbkg* LKO	*Rbpj* LKO
**Metabolism**	−1.79	ns	ns	−0.84	ns
**Metabolism of carbohydrates**	ns	ns	ns	0.45	0.49
Glycosaminoglycan metabolism	ns	ns	ns	0.55	ns
Hyaluronan metabolism	ns	ns	0.29	0.48	0.50
Chondroitin sulfate biosynthesis	ns	ns	0.21	ns	0.36
Chondroitin sulfate/dermatan sulfate metabolism	ns	ns	ns	0.29	ns
Formation of xylulose-5-phosphate	ns	−0.38	−0.16	ns	ns
Fructose biosynthesis	0.33	0.51	ns	0.75	ns
Fructose catabolism	−0.30	ns	ns	ns	ns
Fructose metabolism	ns	ns	ns	0.50	ns
Gluconeogenesis	ns	ns	ns	ns	0.45
Glucose metabolism	ns	ns	ns	ns	0.55
Glycolysis	ns	ns	ns	ns	0.45
Glycogen breakdown (glycogenolysis)	0.17	0.22	ns	ns	ns
Glycogen synthesis	ns	ns	ns	0.18	ns
Pentose phosphate pathway	ns	0.39	ns	ns	ns
**Metabolism of steroids**	ns	ns	ns	−0.72	ns
Metabolism of steroid hormones	ns	ns	ns	−0.24	ns
Androgen biosynthesis	ns	ns	ns	−0.77	−0.90
Glucocorticoid biosynthesis	ns	−0.99	−0.50	−0.84	ns
Mineralocorticoid biosynthesis	ns	−0.99	−0.72	−0.88	ns
Pregnenolone biosynthesis	0.34	0.49	ns	0.41	ns
Estrogen biosynthesis	ns	ns	ns	ns	0.48
Cholesterol biosynthesis	ns	1.73	0.70	ns	ns
Bile acid and bile salt metabolism	ns	ns	−0.20	−0.54	−0.75
Recycling of bile acids and salts	ns	−0.36	−0.29	−0.36	−0.32
Synthesis of bile acids and bile salts	ns	ns	ns	−0.53	−0.69
Synthesis of bile acids and bile salts via 24-hydroxycholesterol	−0.80	ns	−0.22	−0.45	−0.77
Synthesis of bile acids and bile salts via 27-hydroxycholesterol	−0.79	ns	ns	−0.59	−0.96
Synthesis of bile acids and bile salts via 7alpha-hydroxycholesterol	−0.82	ns	ns	−0.72	−1.00
**Fatty acid metabolism**	−1.31	ns	ns	ns	ns
Fatty acyl-CoA biosynthesis	ns	0.81	ns	ns	ns
alpha-linoleic (omega3) and linoleic (omega6) acid metabolism	−0.70	ns	ns	−0.36	−1.49
Mitochondrial Fatty Acid Beta-Oxidation	−1.18	−0.69	−0.46	ns	ns
Mitochondrial fatty acid beta-oxidation of saturated fatty acids	−0.68	−0.41	−0.33	ns	ns
Propionyl-CoA catabolism	−0.37	ns	ns	−0.33	ns
Beta oxidation of decanoyl-CoA to octanoyl-CoA-CoA	−0.56	−0.30	−0.29	ns	ns
Beta oxidation of hexanoyl-CoA to butanoyl-CoA	−0.58	−0.33	−0.27	ns	ns
Beta oxidation of lauroyl-CoA to decanoyl-CoA-CoA	−0.59	−0.37	−0.26	ns	ns
Beta oxidation of octanoyl-CoA to hexanoyl-CoA	−0.57	−0.34	−0.29	ns	ns
Peroxisomal lipid metabolism	−1.12	ns	−0.36	ns	−0.82
Alpha-oxidation of phytanate	−0.61	ns	−0.21	−0.33	ns
Beta-oxidation of pristanoyl-CoA	ns	ns	ns	ns	−0.62
Beta-oxidation of very long chain fatty acids	−0.87	−0.46	−0.38	ns	ns
**Sphingolipid metabolism**	0.49	0.34	ns	0.38	0.77
Glycosphingolipid metabolism	0.56	0.46	ns	0.45	0.76
**Triglyceride metabolism**	ns	ns	0.62	ns	ns
Triglyceride biosynthesis	−0.39	ns	ns	ns	0.54
Triglyceride catabolism	ns	ns	0.92	ns	ns
**Wax and plasmalogen biosynthesis**	ns	0.31	ns	ns	ns
**Ketone body metabolism**	−0.40	ns	ns	ns	−0.43
Synthesis of Ketone Bodies	−0.59	ns	−0.28	−0.22	−0.44
**The citric acid (TCA) cycle and respiratory electron transport**	−1.31	ns	ns	−0.84	ns
Pyruvate metabolism and Citric Acid (TCA) cycle	−0.75	ns	ns	−0.43	ns
**Metabolism of vitamins and cofactors**	−1.35	ns	ns	ns	ns
Metabolism of water-soluble vitamins and cofactors	−1.40	ns	ns	−0.76	ns
Metabolism of fat-soluble vitamins	−0.52	ns	ns	ns	0.43
**Metabolism of amino acids and derivatives**	−1.38	ns	ns	−1.14	ns
Metabolism of amine-derived hormones	−0.43	ns	ns	−0.37	ns
Aspartate and asparagine metabolism	−0.51	ns	ns	−0.22	ns
Branched-chain amino acid catabolism	−0.98	ns	−0.42	−0.73	−0.59
Choline catabolism	ns	ns	ns	−0.32	ns
Degradation of cysteine and homocysteine	−0.59	ns	ns	−0.41	ns
Glutamate and glutamine metabolism	−0.24	ns	ns	ns	ns
Glyoxylate metabolism and glycine degradation	−0.92	ns	−0.43	−0.63	ns
Histidine catabolism	−0.24	−0.27	ns	−0.24	ns
Lysine catabolism	−0.65	ns	−0.25	−0.37	ns
Phenylalanine and tyrosine metabolism	−0.68	ns	ns	−0.25	ns
Phenylalanine metabolism	−0.51	ns	ns	ns	ns
Tyrosine catabolism	−0.44	ns	ns	−0.21	ns
Serine biosynthesis	0.32	ns	ns	ns	ns
Sulfur amino acid metabolism	−0.63	ns	ns	−0.49	ns
Threonine catabolism	−0.43	−0.33	ns	−0.36	ns
Tryptophan catabolism	−0.62	ns	ns	−0.44	ns
Urea cycle	−0.37	ns	ns	−0.29	ns

Presented are log_2_ fold changes of KO vs. WT. ns—non-significant.

**Table 3:** Overview of changes in the level of serum metabolites and in the liver expression of transcription factors in humans with NAFLD and NASH. Selected were metabolites and TFs, which were exposed as key factors in the fibrotic program by genetic mouse models of liver fibrosis. C4: 7-alpha-hydroxy-4-cholesten-3-one.

Factor	Stage of Disease	Change	Reference
**Metabolites**			
Bile acids	NASH	Primary and secondary bile acids are increased	[26]
	NASH	C4 increased	[29]
	NASH	Primary and secondary bile acids increased	[30]
	NASH	Bile acids increase with NASH progression	[31]
	NASH	Bile acids increased	[32]
	NASH	Primary bile acids increased, secondary decreased	[33]
	NAFLD	Total bile acid are decreased but major difference is in composition, bile acid level increases with fibrosis progression	[34]
	NAFLD	Bile acids change with disease progression, direction depended on the type of bile acid	[35]
	NASH	Primary conjugated bile acid increase with fibrosis, unconjugated bile acids decrease	[36]
	NAFLD	Primary and secondary bile acids are increased in higher fibrotic stages, but no change in C4	[27]
Polyunsaturated fatty acids (PUFA)	NAFLD	Decreased	[35]
	Severe NAFLD	Total PUFA decreased in red blood cell membrane, *n*-3 all decreased, *n*-6 majority increased, except linoleic acid decreased	[37]
	NAFLD	Total PUFA *n*-3 decreased in serum	[38]
	NAFLD	Total PUFA decreased in erythrocytes	[39]
	NASH	PUFA (18:3*n*-3) decreased	[9]
	NASH	Eicosapentaenoate (20:5*n*-3), docosahexaenoate (22:6*n*-3), arachidonate (20:4*n*-6) are decreased	[32]
	NASH	PUFA are altered	[40]
Monounsaturated fatty acids (MUFA)	NASH	Total MUFA increased	[40]
	Severe NAFLD	Total MUFA increased in red blood cell membrane	[37]
	NAFLD	Total MUFA increased, docosahexaenoic acid (C22:6) and arachidonic acid (C20:4) decreased in blood	[41]
Sphingolipids	NASH	Sphingomyelin (36:0) increased	[42]
	NAFLD	Sphingomyelins decreased	[43]
	NASH	Sphingomyelin increased	[9]
Ketones	NASH	Decreased	[44]
Branched amino acids	NAFLD	All three increased	[43]
	NASH	All three increased	[32]
**Transcription factors**			
PPARα	NASH	Decreased mRNA expression in liver, negative correlation with NASH progression	[45,46]
LXR, SREBPC1	NAFLD	Increased mRNA and protein expression in liver	[47]
PPARγ	NAFLD	PPARγ2 mRNA is increased in liver	[48]
VDR	steatosis	mRNA is increased in liver	[49]
	NASH	Protein is decreased in liver	[50]

## Data Availability

Data is contained within the article and supplementary material.

## Supplementary Materials

The following are available online at https://www.mdpi.com/1422-0067/22/2/832/s1. Table S1: Characteristics of genetic mouse models of liver fibrosis, Table S2: List of DEGs significant (adjusted *p*-value < 0.05) in at least one genetic model with log2 fold change (KO/WT), Table S3: Numbers of DEGs, KEGG pathways, Reactome pathways and overlap between different genetic models of liver fibrosis, Table S4: Enriched KEGG pathways significant (adjusted *p*-value < 0.05) in at least one genetic model with log2 fold change (KO/WT), Table S5: Enriched Reactome pathways significant (adjusted *p*-value < 0.05) in at least one genetic model with log2 fold change (KO/WT), Table S6: Comparison of enriched KEGG pathways between genetic mouse models with pathways reported for NAFLD and NASH in Teufel A. et al. 2016, Table S7: Enriched transcription factors significant (adjusted *p*-value < 0.05) in at least one genetic model with log2 fold change (KO/WT), Table S8: Mouse liver samples that were included in the study, Figure S1: A heat map of 62 common DEGs in genetic mouse models of liver fibrosis. Columns were scaled to have mean zero and standard deviation one.

## Abbreviations

AASLD	American Association for the Study of Liver Diseases
AMPK	AMP-activated protein kinase
CYP51	Cytochrome P450, family 51
DEGs	Differentially expressed genes
E2F1	E2F transcription factor 1
EASL	The European Association for the Study of the Liver
ECM	Extracellular matrix
ERα	Estrogen receptor alpha
ErbB	Erb-B2 receptor tyrosine kinase
F	Female
FXR	Farnesoid X receptor
GEM	Genome-scale metabolic model
GLMP	Glycosylated lysosomal membrane protein
HCC	Hepatocellular carcinoma
HDL	High-density lipoprotein
IGF1R	Insulin like growth factor 1 receptor
IKBKG	Inhibitor of nuclear factor kappa B kinase regulatory subunit gamma
KEGG	Kyoto Encyclopedia of Genes and Genomes
KO	Knock-out
LKO	Liver knock-out
LXR	Liver X receptor
M	Male
MAFLD	Metabolic Associated Fatty Liver Disease
MAPK	Mitogen-activated protein kinase
MFSD1	Major facilitator superfamily domain containing 1
mTOR	The mammalian target of rapamycin
NAFLD	Non-alcoholic fatty liver disease
NASH	Non-alcoholic steatohepatitis
NF-κB	Nuclear factor kappa-light-chain-enhancer of activated B cells
pGSEA	Parametric gene set enrichment analysis
PPAR	Peroxisome proliferator activated receptor
RBPJ	Recombination signal binding protein for immunoglobulin kappa J region
RXR	Retinoid X receptor
SREBP1	Sterol regulatory element-binding protein 1
STRING	Search tool for recurring instances of neighboring genes
TAG	Triglycerides
TCA	Tricarboxylic acid cycle
TF	Transcription factor
TGF-β	Transforming growth factor beta
TGFBI	Transforming growth factor beta induced
TRANSFAC	TRANScription FACtor
VDR	Vitamin D receptor
VEGF	Vascular endothelial growth factor
WT	Wild type
