# Supplementary figures and images for: Common Transcriptional Program of Liver Fibrosis in Mouse Genetic Models and Humans
Source: Int J Mol Sci. 2021 Jan 15;22(2):832. doi: 10.3390/ijms22020832 (PMC7830925; doi:10.3390/ijms22020832)

A heat map of 62 common DEGs

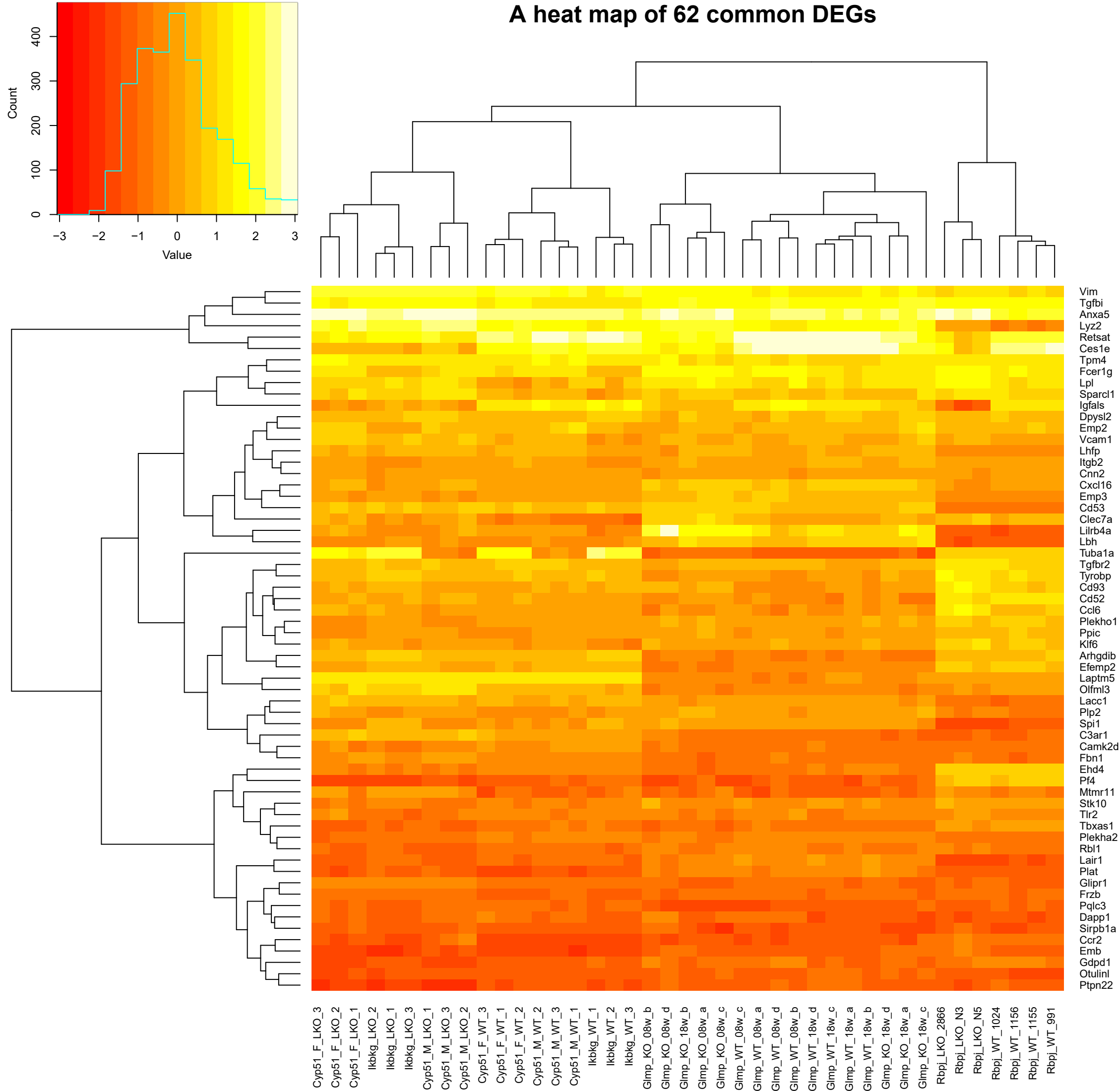

Supplement: Supplementary file 1 [file ijms-22-00832-s001.zip › Supplementary Figure S1.pdf]
